# Supplementary material for: T cell toxicity induced by tigecycline binding to the mitochondrial ribosome
Source: Nat Commun. 2025 May 1;16:4080. doi: 10.1038/s41467-025-59388-9 (PMC12045974; doi:10.1038/s41467-025-59388-9)
Supplement: Supplementary file 1 — Supplementary Information [file 41467_2025_59388_MOESM1_ESM.pdf]

Supplementary information

T cell toxicity induced by tigecycline binding to the mitochondrial ribosome

.

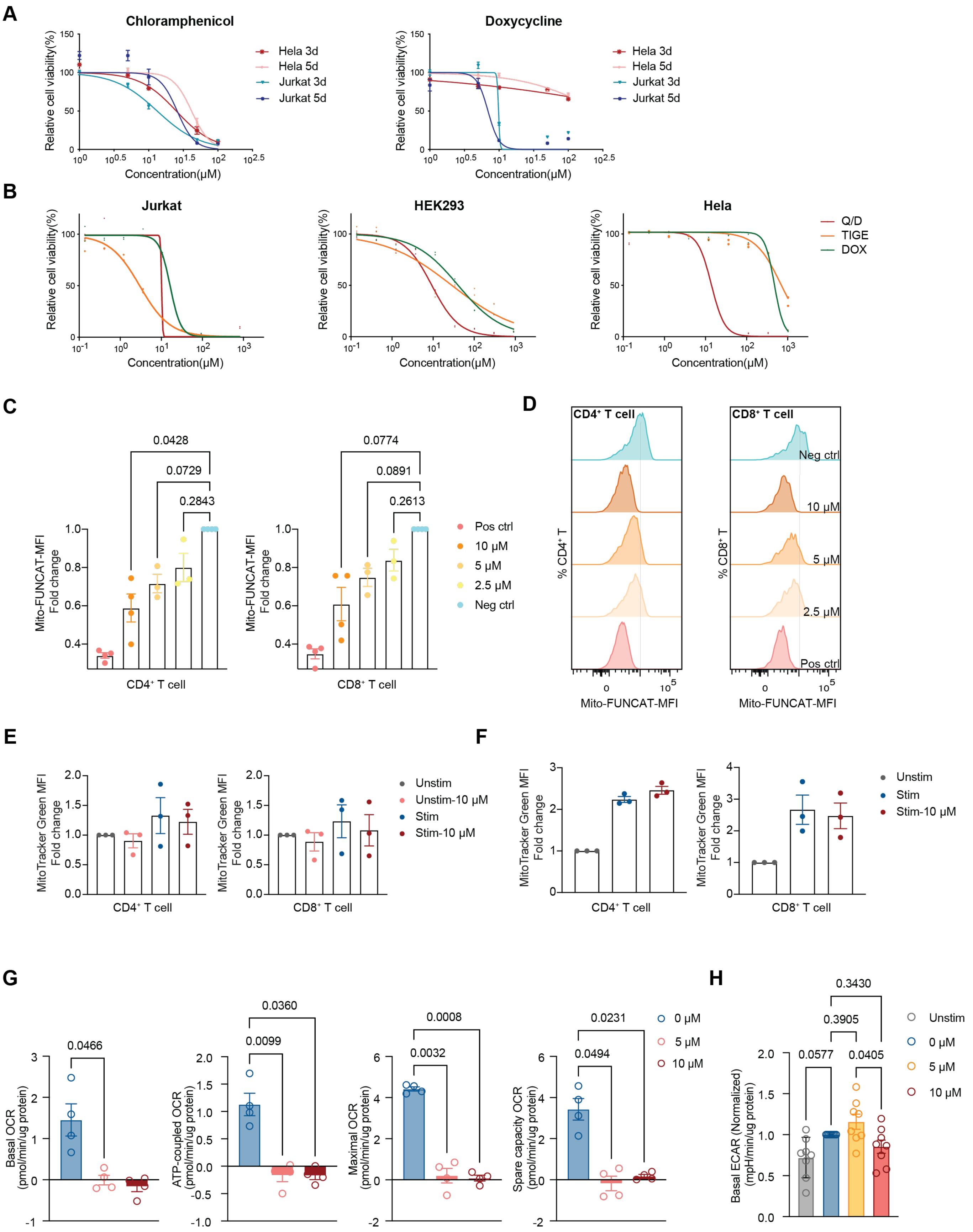
A
Chloramphenicol
Doxycycline
Relative cell viability(%)
Concentration(μM)
Hela 3d
Hela 5d
Jurkat 3d
Jurkat 5d
B
Jurkat
HEK293
Hela
Relative cell viability(%)
Concentration(μM)
Q/D
TIGE
DOX
C
Mito-FUNCAT-MFI Fold change
0.0428
0.0729
0.2843
CD4⁺ T cell
0.0774
0.0891
0.2613
CD8⁺ T cell
Pos ctrl
10 μM
5 μM
2.5 μM
Neg ctrl
D
CD4⁺ T cell
CD8⁺ T cell
% CD4⁺ T
% CD8⁺ T
Neg ctrl
10 μM
5 μM
2.5 μM
Pos ctrl
Mito-FUNCAT-MFI
E
MitoTracker Green MFI Fold change
CD4⁺ T cell
CD8⁺ T cell
Unstim
Unstim-10 μM
Stim
Stim-10 μM
F
MitoTracker Green MFI Fold change
CD4⁺ T cell
CD8⁺ T cell
Unstim
Stim
Stim-10 μM
G
Basal OCR (pmol/min/ug protein)
0.0466
ATP-coupled OCR (pmol/min/ug protein)
0.0360
0.0099
Maximal OCR (pmol/min/ug protein)
0.0008
0.0032
Spare capacity OCR (pmol/min/ug protein)
0.0231
0.0494
0 μM
5 μM
10 μM
H
Basal ECAR (Normalized) (mpH/min/ug protein)
0.3430
0.3905
0.0577
0.0405
Unstim
0 μM
5 μM
10 μM

Supplementary Fig. 1. Effects of selected antibiotics on cell viability and mitochondrial function.

(A) Representative dose-response curves of chloramphenicol and doxycycline cytotoxicity on Jurkat T and Hela cell lines for 3 or 5 days. n = 5 biological replicates, mean ± SEM. Source data are provided as a Source Data file.

(B) Representative dose-response curves of tigecycline, doxycycline, and Q/D cytotoxicity on Jurkat, Hek293, and Hela cells measured 72 h after treatment. n =2 biological replicates, both replicates are shown. Source data are provided as a Source Data file.

(C-D) Mito-FUNCAT-FACS assay of mitochondrial translation on activated T cells. After being stimulated for 48 h, purified T cells were treated with anisomycin to inhibit cytosolic translation (negative control), followed by treatment with either chloramphenicol (309 µM, positive control) or varying concentrations of tigecycline (2.5-10 µM) for 30 min. Mitochondrial nascent proteins were specifically labeled with a methionine analog, L-homopropargylglycine (HPG) for 3h. **(C)** Quantification of mitochondrial translation (expressed as fold change relative to negative control) in $CD4^+$ and $CD8^+$ T cells, respectively. **(D)** Representative representative flow cytometry histograms of AF488-conjugated HPG signals in $CD4^+$ and $CD8^+$ T cell populations under different treatment conditions. n = 3 or 4 biological replicates, mean ± SEM. Source data are provided as a Source Data file.

(E-F) Quantification of mitochondrial mass in $CD4^+$ and $CD8^+$ T cell after treatment with 10 µM Tigecycline for (E) 24 h or (F) 7 days (expressed as fold change relative to untreated control). n = 3 biological replicates, mean ± SEM. Source data are provided as a Source Data file.

(G-H) Seahorse Mito Stress Test quantification of **(G)** basal, ATP-coupled, maximal, spare capacity OCR and **(H)** basal ECAR in healthy donor PBMCs cultured with or without tigecycline for 6 days after stimulation with anti-CD3/CD28 antibodies + IL-2 (10ng/ml). Corresponding to Figure 1E. Basal ECAR was normalized to stimulated control. n = 4 - 8 biological replicates, mean ± SEM. An RM one-way ANOVA with Tukey's multiple comparisons test was used to analyze the data. Source data are provided as a Source Data file.

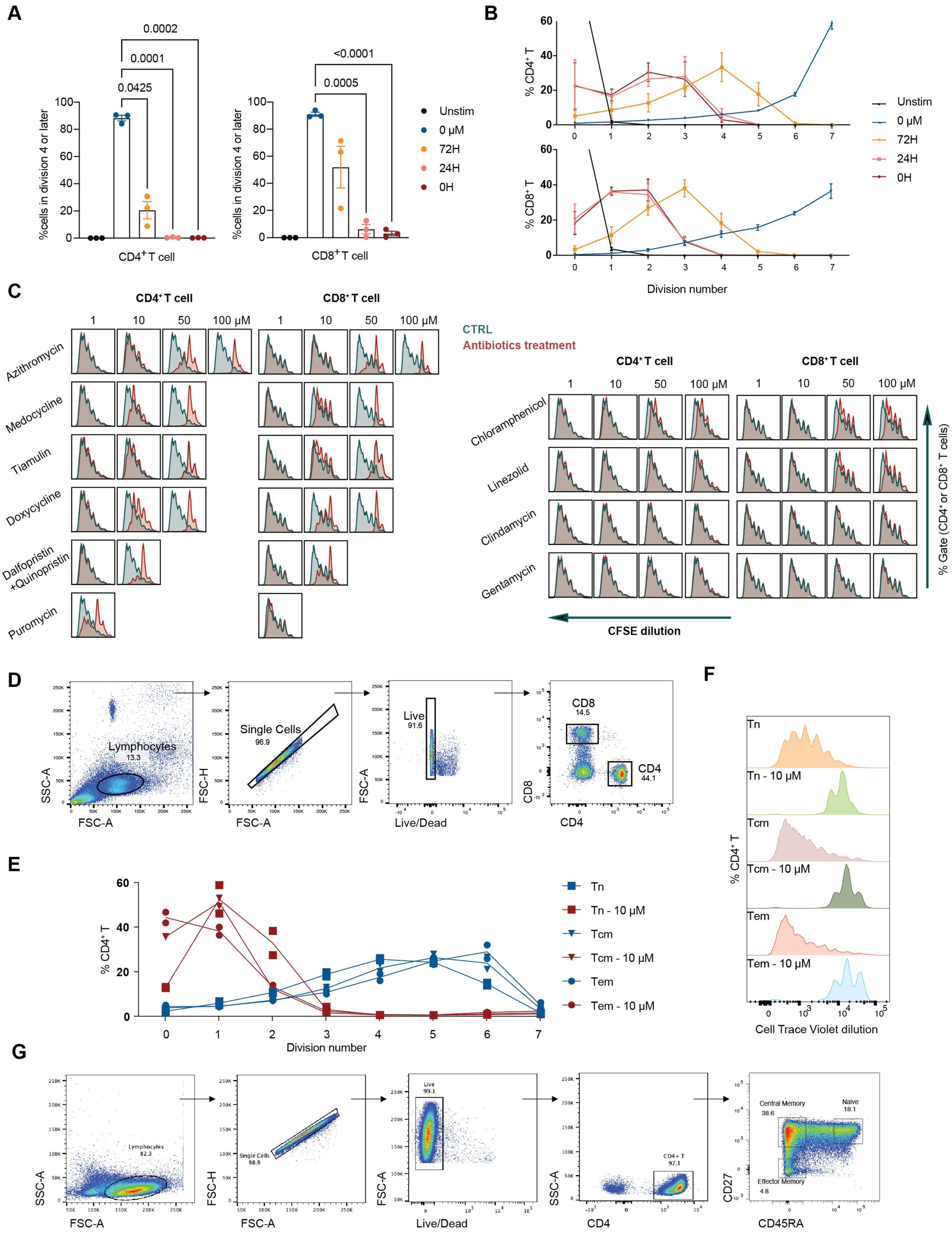
A
0.0002
0.0001
0.0425
%cells in division 4 or later
CD4+ T cell
<0.0001
0.0005
CD8+ T cell
Unstim
0 μM
72H
24H
0H
B
% CD4+ T
% CD8+ T
Division number
C
CD4+ T cell
CD8+ T cell
1
10
50
100 μM
Azithromycin
Medocycline
Tiamulin
Doxycycline
Dalfopristin +Quinopristin
Puromycin
CTRL
Antibiotics treatment
Chloramphenicol
Linezolid
Clindamycin
Gentamycin
% Gate (CD4+ or CD8+ T cells)
CFSE dilution
D
Lymphocytes
13.3
Single Cells
96.9
Live
91.6
CD8
14.5
CD4
44.1
SSC-A
FSC-A
FSC-H
Live/Dead
F
Tn
Tn - 10 μM
Tcm
Tcm - 10 μM
Tem
Tem - 10 μM
% CD4+ T
Cell Trace Violet dilution
E
Division number
G
Lymphocytes
82.3
Single Cells
98.9
Live
99.1
CD4+ T
97.1
Central Memory
38.6
Naive
18.1
Effector Memory
4.8
CD27
CD45RA

Supplementary Fig. 2. Analysis of T cell subsets proliferation and response to antibiotics.

(A-B) T cell proliferation assessed by flow cytometry. PBMC T cells were stimulated with anti-CD3/CD28 beads + IL-2 (10ng/ml) and proliferation was assessed by CTV dilution in live cells after 6 days (*n* = 3 blood donor PBMC samples). T cells were treated with 10 µM tigecycline at 0, 24 or 72 h post activation. T cells were labelled with CTV prior to stimulation. Source data are provided as a Source Data file.

(A) Percentage of live T cells in division four or later, mean ± SEM. An RM one-way ANOVA with Tukey's multiple comparisons test was applied to analyze the data.

(B) Percentage of live $CD4^+$ and $CD8^+$ T cells in each division. Source data are provided as a Source Data file.

(C) Proliferation profiles of $CD4^+$ and $CD8^+$ T cells from PBMCs treated with antibiotics for 6 days post-stimulation with anti-CD3/CD28 beads + IL-2 (10ng/ml). PBMCs were labeled with CFSE prior to stimulation. The histogram plots show an overlay of CFSE dilutions after 6 days. For some antibiotics, higher concentrations led to cell death, which is indicated by the lack of proliferation profiles. Source data are provided as a Source Data file.

(D) Gating strategy for the flow experiments in Figure 1F, 1G-H and Supplementary Figure 1C-D, 2A-C.

(E-G) Proliferation of FACS-isolated $CD4^+$ T cell subsets. Tn, Tcm and Tem $CD4^+$ T cells were sorted from purified total $CD4^+$ T cells isolated by magnetic separation. Tn was defined as Live $CD4^+CD45RA^+CD27^+$; Tcm as Live $CD4^+CD45RA^-CD27^+$; Tem as Live $CD4^+CD45RA^-CD27^-$. **(E)** Percentage of live Tn, Tcm and Tem cells in each division. n=2 biological replicates, both replicates are shown. **(F)** Representative CTV dot plots from non-treated and 10 µM tigecycline-treated samples. **(G)** Representative profiles of donor samples, corresponding to Figure 1I-J. Source data are provided as a Source Data file.

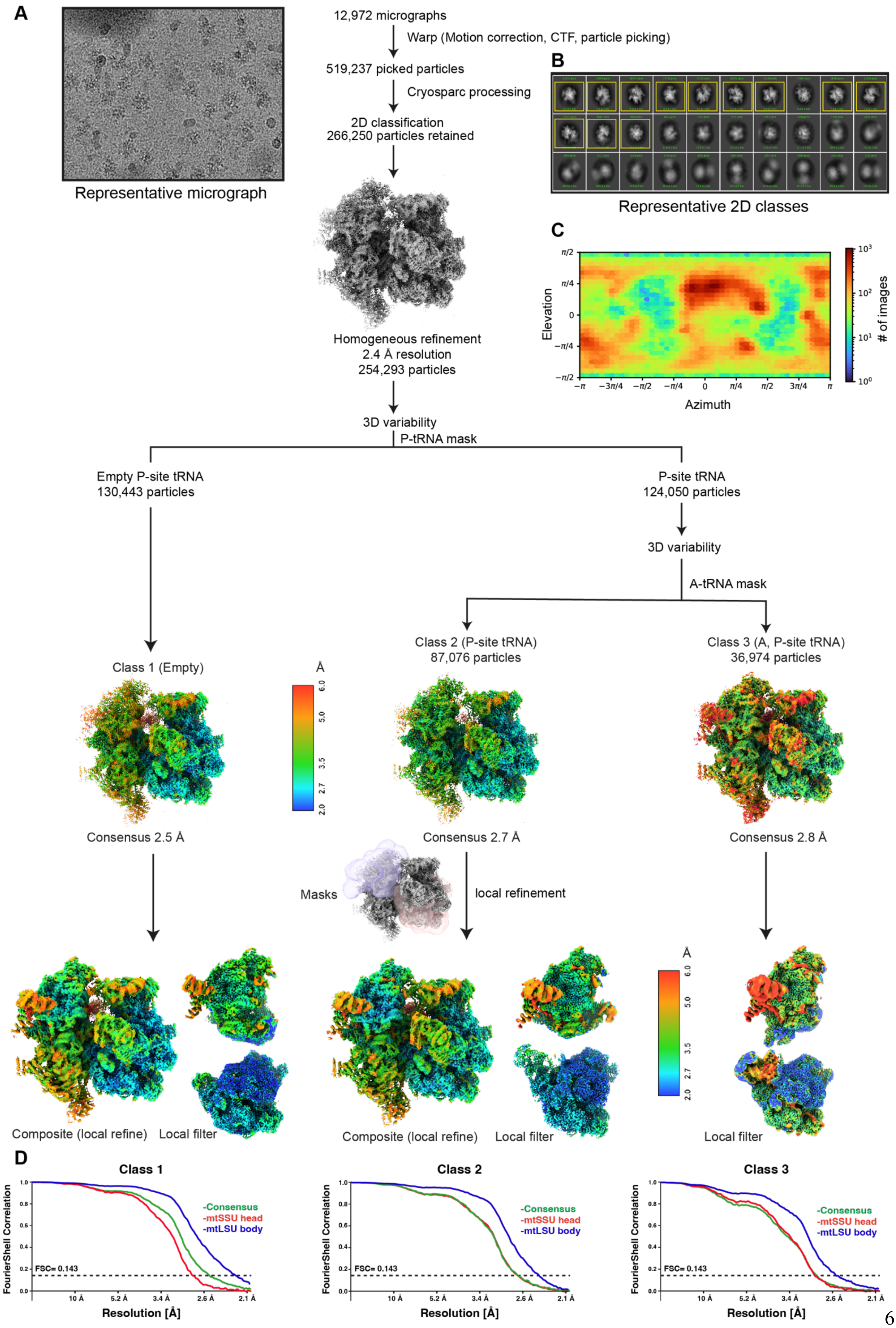

Gold-standard FSC curves of the 3D reconstructions

Supplementary Fig. 3. Overview of cryo-EM data processing.

Cryo-EM data processing workflow for the tigecycline-bound mitoribosome complex. Data processing scheme illustrates 3D classification through homogenous refinement resulting in three classes of tigecycline-bound monosomes.

(A) Representative micrograph.

(B) 2D class averages.

(C) Heatmap of the angular distribution for particle projections after homogenous refinement calculated in CryoSPARC.

(D) Masked Fourier Shell Correlation (FSC) curve of the half maps and local-masked refinement

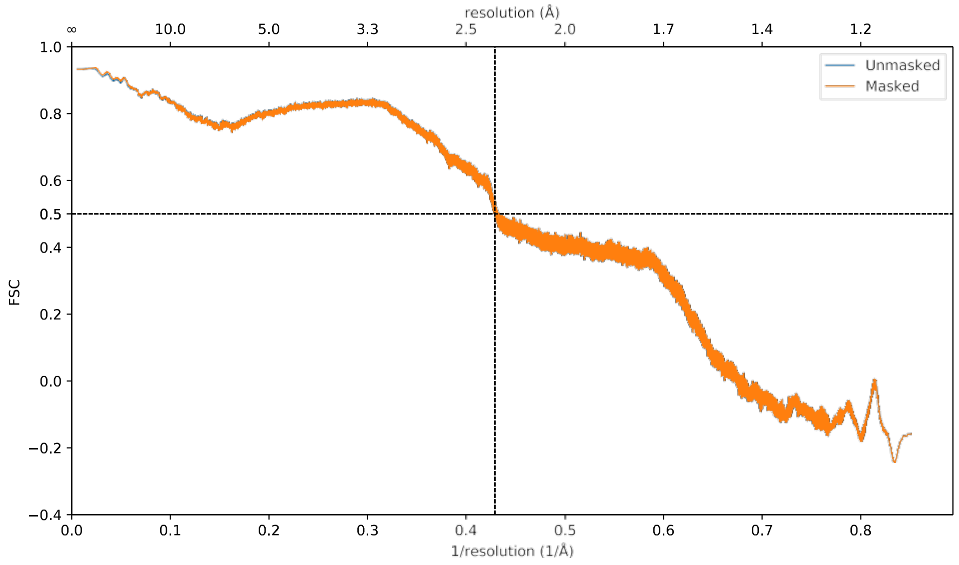

Supplementary Fig. 4. Map-model FSC.

Map-model FSC curves with and without mask.

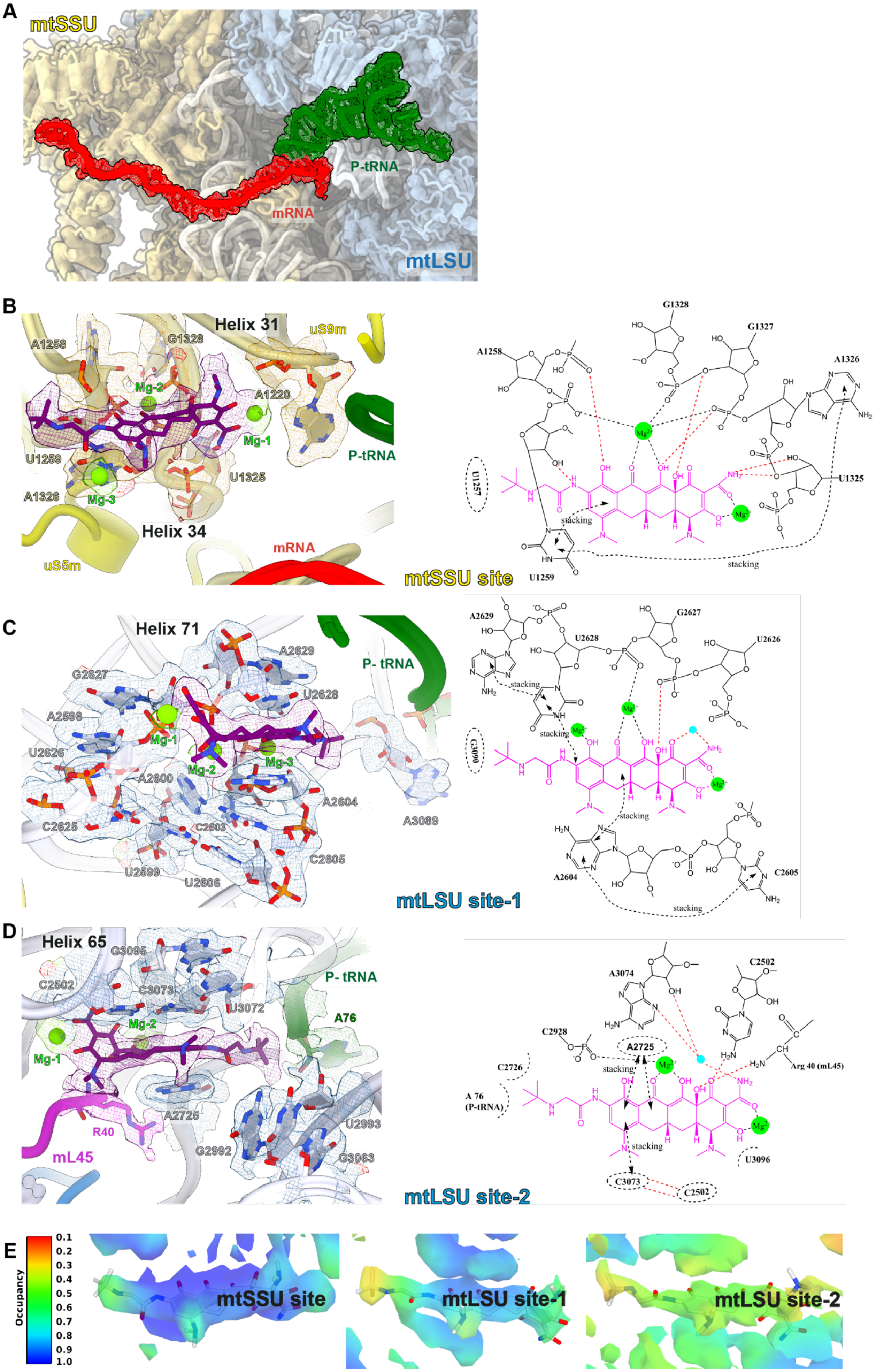
A
mtSSU
P-tRNA
mRNA
mtLSU
B
Helix 31
uS9m
A1258
G1328
Mg-2
A1220
Mg-1
P-tRNA
U1259
U1325
A1326
Mg-3
Helix 34
uS5m
mRNA
mtSSU site
C
Helix 71
A2629
P- tRNA
G2627
U2628
A2598
Mg-1
U2626
Mg-2
Mg-3
A2600
A2604
A3089
C2625
C2603
C2605
U2599
U2606
mtLSU site-1
D
Helix 65
G3095
C2502
G3073
P- tRNA
U3072
Mg-2
A76
Mg-1
A2725
R40
U2993
mL45
G2992
G3063
mtLSU site-2
E
Occupancy
0.1
0.2
0.3
0.4
0.5
0.6
0.7
0.8
0.9
1.0
mtSSU site
mtLSU site-1
mtLSU site-2

Supplementary Fig. 5. CryoEM densities, surrounding environment and schematic representation of the interactions of tigecycline on mitoribosome.

(A) Map-to-model fitting for mRNA and tRNA.

(B-D) The electron density map and schematic interactions for the three binding sites. **(B)** mtSSU site; **(C)** mtLSU site-1; and **(D)** mtLSU site-2 of the tigecycline at the mtSSU and mtLSU. Carved densities are also shown for the nucleotide residues that are involved in interactions with the tigecycline together with the Mg^{2+} ion complexes. The density maps (left panels) were colored corresponding to the model. For the schematic interactions (right panels), the hydrogen bonds and indirect bonds via Mg^{2+} ion between mitoribosome and tigecycline are shown as red dash line and black dash line, respectively. The stacking of the bases and the drug is shown as a black dashed line with 2 arrowheads. Putative Mg^{2+} ions and water molecules were shown as green and cyan, respectively.

(E) The occupancy of tigecycline on mtSSU site, mtLSU site-1 and mtLSU site-2 have been shown based on the density analysis done by OccuPy[1].

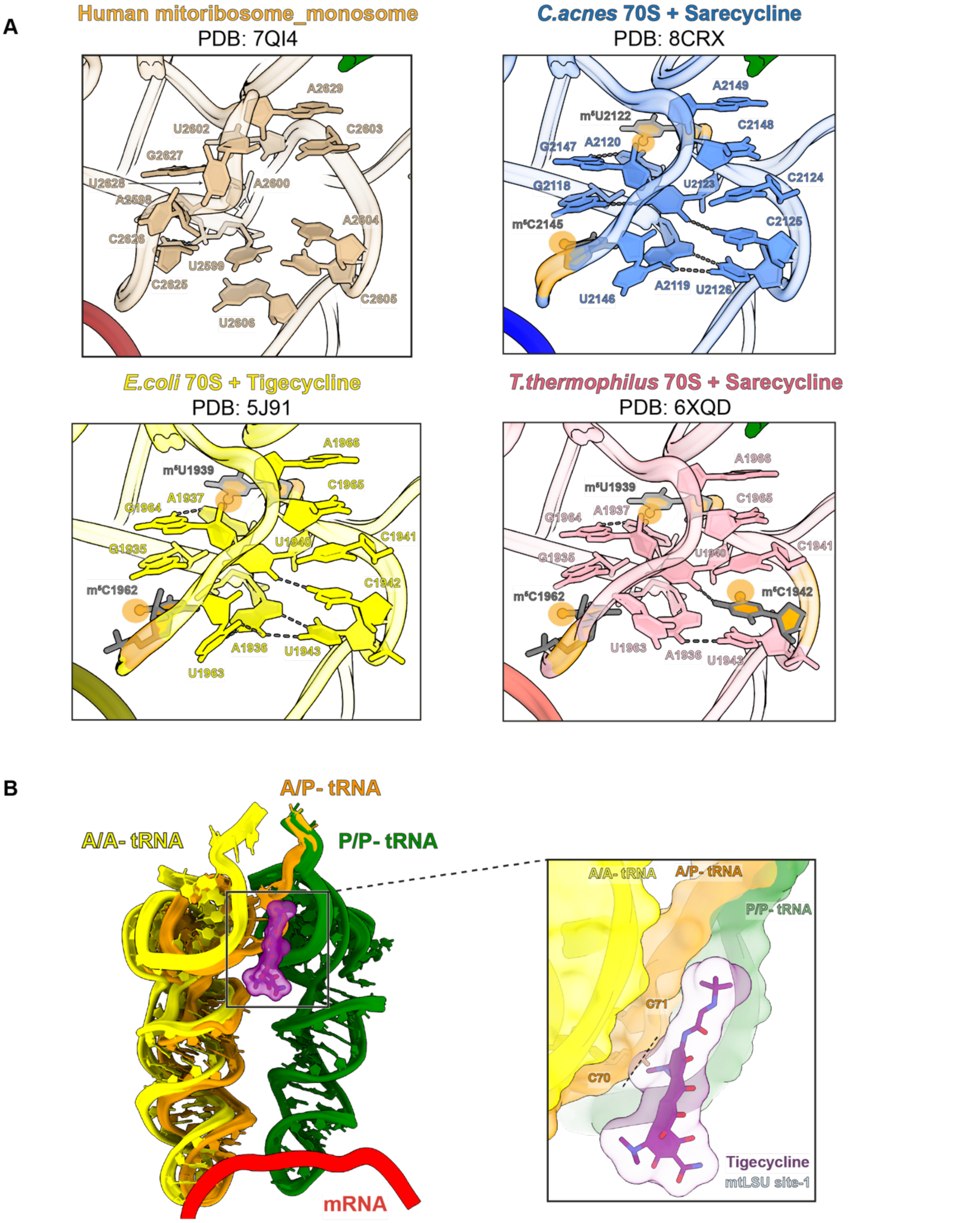

Supplementary Fig. 6. Comparison of the mtLSU site-1 with its bacterial counterparts and functional implication of mtLSU site-1 on inhibiting protein translation of human mitoribosome.

(A) Unlike helix 71 in mammalian mitoribosome (here, tigecycline bound), in bacteria, helix 71 forms multiple intermolecular hydrogen bonds between nucleotides. Furthermore, the helices are surrounded by methylations such as m5U1939 and m5C1962 (highlighted in orange) in *E.coli* (PDB: 5J91[2]), *T.therомphilus* (PDB: 6XQD[3]), and *C.acnes* (PDB: 8CRX[4]) (equivalent

m5U2122 and m5C2145 in *C.acnes*). These methylations likely contribute to the rigidity of the region, enhancing its structural stability. *T.theromphilus* carries an additional methylation at cytosine m5C1942. The observed methylations in bacteria are absent in mitoribosomal 16S rRNA.

(B) The position of mtLSU site-1 in relation to P/P-tRNA (light green, this study), A/P-tRNA (cyan, PDB: 7QI6[5]) and A/A-tRNA (light blue, this study). The tigecycline is shown as both a stick (purple) and a transparent sphere. The insertion represents a close view to show the clash of the drug with the phosphate backbone of C71 and the OH group of the ribose ring of C70 in A/P-tRNA (cyan).

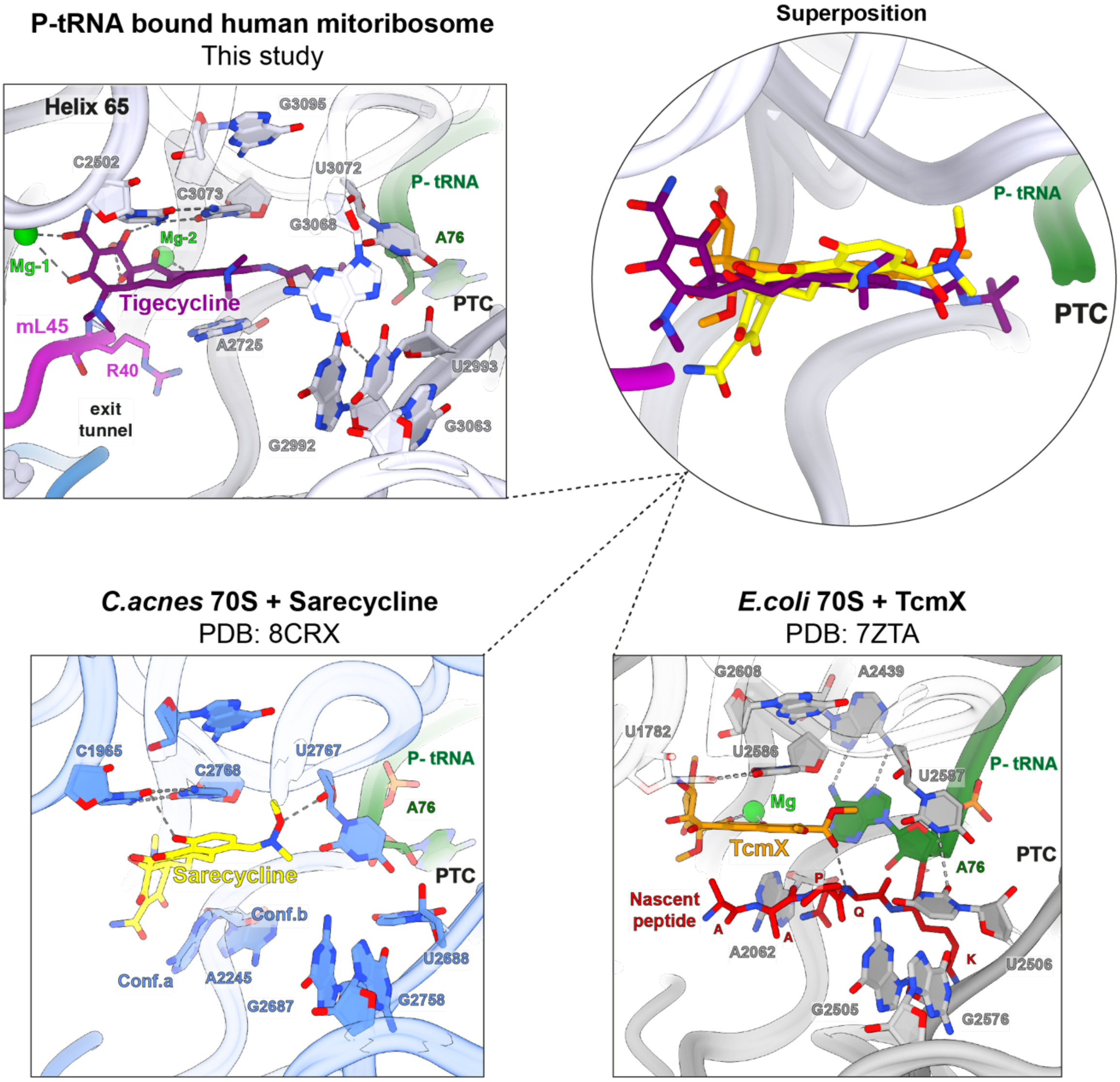

Supplementary Fig. 7. Binding of antibiotics to the PTC of ribosome.

The comparison of the binding of the antibiotics: tigecycline (purple), sarecycline (yellow; PDB:8CRX[4]) and TcmX (orange; PDB:7ZTA[6]) in the PTC of the corresponding ribosomes.

Supplementary Table 1

**Data collection and processing**	***Class 1 (Empty)***	***Class 2 (P-site tRNA)***	***Class 3 (A, P-site tRNA)***
Microscope	Titan Krios		
Detector	K3		
Magnification	165,000		
Voltage [kV]	300		
Total electron exposure [$e^-/Å^2$]	45		
Defocus range [µm]	−0.4 to −1.6		
Pixel size [Å]	0.505		
Symmetry imposed	C_1		
Processed particles (no.)	266,250		
Final particles (no.)	130,443	87,076	36,974
Map resolution [Å] (overall/SSU-head/LSU-body)	2.5/2.8/2.2	2.7/2.7/2.4	2.8/2.9/2.5
FSC threshold	0.143	0.143	0.143
Map resolution range [Å]	2–6	2–6	2–6
Map-sharpening *B*-factor (overall/SSU-head/LSU-body)	-28.9/-44.4/-31.2	-33.6/-37.5/-37.2	-27.2/-31.3/-31.1
**Refinement**			
Model composition			
Total atoms (non-hydrogen/hydrogen)		323,256/147,806	
Chains (RNA/protein)		89	
RNA residues		2,684	
Protein residues		14,365	
Metal ions (Mg^{2+}/K^+)		94/16	
Ligands (2Fe–2S/ATP/T1C)		3/1/3	
Waters		974	
Model to map CC ($CC_{mask}/CC_{box}/CC_{peaks}/CC_{volume}$)		0.78/0.82/0.76/0.77	
Resolution [Å] by model-to-map FSC, threshold 0.50 (masked/unmasked)		2.33/2.33	
Average *B*-factor (RNA/protein/metal ion and ligand/water)		21.04/24.67/23.07/15.6	
R.m.s. deviations, bond lengths [Å]/bond angles [°]		0.007/0.683	
**Validation**			
Clash score		2.72	
Rotamer outliers [%]		0	
Ramachandran plot [%] (favored/allowed/disallowed)		97.57/2.41/0.02	
CaBLAM outliers [%]		1.04	
C_β outliers [%]		0	
MolProbity score		1.15	
PDB/EMDB accession code (Consensus/SSU-head/LSU-body/Composite)	EMD-19544/19545/19546	8RRI/EMD-19493/19490/19491/19460	EMD-19526/19539/19542

Supplementary Table 1. Cryo-EM data collection, processing, model refinement, and validation statistics.

Supplementary Table 2

Antibodies		
Total OXPHOS Human WB Antibody Cocktail	Abcam	Ab110411
MRPL37/mL37	Sigma	HPA025826
MRPS15/uS15m	Proteintech Group	17006-1
HSP60	Enzo Lifesciences	AB1-SPA-807-E
b-actin	Abcam	Ab8224
GAPDH	Abcam	Ab8245
HRP secondary rabbit	GE Healthcare	NA9340V
HRP secondary mouse	GE Healthcare	NA9310V
Human CD3	BD Biosciences	Clone UCHT1, APC
Human CD4	BD Biosciences	Clone RPA-T4, AF700
Human CD8	BD Biosciences	Clone RPA-T8, PerCP
Human CD45RA	BD Biosciences	Clone HI100, PE/Dazzle
Human CD27	BD Biosciences	Clone M-T271, PE/Cyanine 7
Human CD25	BD Biosciences	Clone M-A251, PE

Supplementary Table 2. List of antibodies used in the study.

Supplementary References

1. Forsberg, B. O., Shah, P. N. M. & Burt, A. A robust normalized local filter to estimate compositional heterogeneity directly from cryo-EM maps. *Nat Commun* **14**, (2023).
2. Cocozaki, A. I. *et al.* Resistance mutations generate divergent antibiotic susceptibility profiles against translation inhibitors. *Proc Natl Acad Sci U S A* **113**, 8188–8193 (2016).
3. Batool, Z., Lomakin, I. B., Polikanov, Y. S. & Bunick, C. G. Sarecycline interferes with tRNA accommodation and tethers mRNA to the 70S ribosome. *Proc Natl Acad Sci U S A* **117**, 20530–20537 (2020).
4. Lomakin, I. B., Devarkar, S. C., Patel, S., Grada, A. & Bunick, C. G. Sarecycline inhibits protein translation in Cutibacterium acnes 70S ribosome using a two-site mechanism. *Nucleic Acids Res* **51**, 2915–2930 (2023).
5. Singh, V. *et al.* Mitoribosome structure with cofactors and modifications reveals mechanism of ligand binding and interactions with L1 stalk. *Nat Commun* **15**, 1–22 (2024).
6. Leroy, E. C., Perry, T. N., Renault, T. T. & Innis, C. A. Tetracenomycin X sequesters peptidyl-tRNA during translation of QK motifs. *Nat Chem Biol* **19**, 1091–1096 (2023).
