# Supplementary material for: T cell toxicity induced by tigecycline binding to the mitochondrial ribosome
Source: Nat Commun. 2025 May 1;16:4080. doi: 10.1038/s41467-025-59388-9 (PMC12045974; doi:10.1038/s41467-025-59388-9)
Supplement: Supplementary file 2 — Reporting Summary [file 41467_2025_59388_MOESM2_ESM.pdf]

nature portfolio

Corresponding author(s): Xaquin Castro Dopico
Joanna Rorbach

Last updated by author(s): March 24, 2025

Reporting Summary

Nature Portfolio wishes to improve the reproducibility of the work that we publish. This form provides structure for consistency and transparency in reporting. For further information on Nature Portfolio policies, see our Editorial Policies and the Editorial Policy Checklist.

Statistics

For all statistical analyses, confirm that the following items are present in the figure legend, table legend, main text, or Methods section.

n/a	Confirmed	
☐	☒	The exact sample size (*n*) for each experimental group/condition, given as a discrete number and unit of measurement
☐	☒	A statement on whether measurements were taken from distinct samples or whether the same sample was measured repeatedly
☐	☒	The statistical test(s) used AND whether they are one- or two-sided<br>*Only common tests should be described solely by name; describe more complex techniques in the Methods section.*
☒	☐	A description of all covariates tested
☐	☒	A description of any assumptions or corrections, such as tests of normality and adjustment for multiple comparisons
☐	☒	A full description of the statistical parameters including central tendency (e.g. means) or other basic estimates (e.g. regression coefficient) AND variation (e.g. standard deviation) or associated estimates of uncertainty (e.g. confidence intervals)
☐	☒	For null hypothesis testing, the test statistic (e.g. *F*, *t*, *r*) with confidence intervals, effect sizes, degrees of freedom and *P* value noted<br>*Give P values as exact values whenever suitable.*
☒	☐	For Bayesian analysis, information on the choice of priors and Markov chain Monte Carlo settings
☒	☐	For hierarchical and complex designs, identification of the appropriate level for tests and full reporting of outcomes
☒	☐	Estimates of effect sizes (e.g. Cohen's *d*, Pearson's *r*), indicating how they were calculated

Our web collection on statistics for biologists contains articles on many of the points above.

Software and code

Policy information about availability of computer code

Data collection	Cell viability data were collected on Tecan plate reader.<br>Western blot data were collected with Clarity Western ECL Substrate (Bio-Rad).<br>Extracellular Metabolic Flux data were collected on Seahorse XFe96 Analyzer (Seahorse Bioscience).<br>De novo mitochondrial translation data were collected on Typhoon FLA 7000 Phosphorimager (GE Healthcare).<br>Flow cytometry data were collected on BD Celesta and Aria Fusion instruments.<br>Cryo-EM data were collected with a Krios G3i electron microscope (Thermofisher) operated at 300 kV and equipped with a K3 Bioquantum detector (Gatan). Automated data collection software was used during collection (EPU 2, Thermofisher) which was performed at 165,000x EFTEM SA magnification, yielding a calibrated pixel size of 0.505 A.
Data analysis	Image analysis was performed in Image Lab 6.0. l.<br>Flow cytometry data were analyzed in FlowJo 10.8.2 (BD Biosciences).<br>Statistical analyses were carried out in Prism 9 (GraphPad).<br>For cryoEM data, Motion correction, CTF-estimation, Fourier cropping (to 1.01 A/px), picking and extraction in 512 pixel boxes were performed on the fly using WARP 1.0.9. Cryosparc v4 was used to run 2D classification and further analyses.

For manuscripts utilizing custom algorithms or software that are central to the research but not yet described in published literature, software must be made available to editors and reviewers. We strongly encourage code deposition in a community repository (e.g. GitHub). See the Nature Portfolio guidelines for submitting code & software for further information.

Data

Policy information about availability of data

All manuscripts must include a data availability statement. This statement should provide the following information, where applicable:

- Accession codes, unique identifiers, or web links for publicly available datasets
- A description of any restrictions on data availability
- For clinical datasets or third party data, please ensure that the statement adheres to our policy

Cryo-EM maps have been deposited at the Electron Microscopy Data Bank as follows: Class 1 (empty class), EMD-19544 (consensus map), EMD-19545 (SSU-head), EMD-19546 (LSU-body); Class 2 (P-site tRNA), EMD-19493 (consensus map), EMD-19490 {SSU-head), EMD-19491 (LSU-body), EMD-19460 (composite); Class 3 (A- and P-site tRNA), EMD-19526 (consensus map), EMD-19539 (SSU-head), EMD-19542 (LSU-body), EMD-19460 (composite map). Associated molecular model has been deposited at the PDB: 8RRI (tigecycline bound human mitoribosome containing P-site tRNA and mRNA). The map and the model are available at https://figshare.com/; Login: anas.khawaja@ki.se; Password: AntibioticTigl2##. Source data are provided with this paper.

Research involving human participants, their data, or biological material

Policy information about studies with human participants or human data. See also policy information about sex, gender (identity/presentation), and sexual orientation and race, ethnicity and racism.

Reporting on sex and gender	Human peripheral blood mononuclear cells (PBMCs) were obtained from anonymized blood donor samples at Karolinska University Hospital: metadata not available.
Reporting on race, ethnicity, or other socially relevant groupings	As above.
Population characteristics	As above.
Recruitment	Buffy coats were delivered by the blood donor center and PBMCs isolated using standard methods.
Ethics oversight	Ethical permit not required.

Note that full information on the approval of the study protocol must also be provided in the manuscript.

Field-specific reporting

Please select the one below that is the best fit for your research. If you are not sure, read the appropriate sections before making your selection.

☒ Life sciences ☐ Behavioural & social sciences ☐ Ecological, evolutionary & environmental sciences

For a reference copy of the document with all sections, see nature.com/documents/nr-reporting-summary-flat.pdf

Life sciences study design

All studies must disclose on these points even when the disclosure is negative.

Sample size	Sample size has been disclosed in this manuscript. Sample sizes were determined based on field standards for obtaining sufficient statistical power (at least 3 biological replicates).
Data exclusions	No data were excluded.
Replication	Attempts to replicate cellular phenotypes were successful.
Randomization	Anonymized human blood donors were used for cellular studies.
Blinding	Blinding was not relevant to this study.

Reporting for specific materials, systems and methods

We require information from authors about some types of materials, experimental systems and methods used in many studies. Here, indicate whether each material, system or method listed is relevant to your study. If you are not sure if a list item applies to your research, read the appropriate section before selecting a response.

Materials & experimental systems

n/a	Involved in the study
☐	☒ Antibodies
☐	☒ Eukaryotic cell lines
☒	☐ Palaeontology and archaeology
☒	☐ Animals and other organisms
☒	☐ Clinical data
☒	☐ Dual use research of concern
☒	☐ Plants

Methods

n/a	Involved in the study
☒	☐ ChIP-seq
☐	☒ Flow cytometry
☒	☐ MRI-based neuroimaging

Antibodies

Antibodies used

Total OXPHOS Human WB Antibody Cocktail (Abeam) Ab110411 1:1000
Anti-human HSP60 (Enzo Lifesciences) ABl-SPA-807-E 1:1000
Anti-human GAPDH (Abeam) Ab8245 1:1000
Anti-human Beta-actin (Abeam) Ab8224 1:1000
Anti-human CD3 (BD Biosciences) UCHT1 300411 1:200
Anti-human CD4 (BD Biosciences) RPA-T4 300526 1:200
Anti-human CD8 (BD Biosciences) RPA-T8 301029 1:200
Anti-human CD27 (BD Biosciences) M-T271 560609 1:200
Anti-human CD45RA (BD Biosciences) Hll00 562326 1:200
Anti-human CD25 (BD Biosciences) M-A251 356103 1:200
HRP secondary rabbit (GE Healthcare) NA9340V 1:3000
HRP secondary mouse (GE Healthcare) NA9310V 1:3000

Validation

OXPHOS Human WB Antibody Cocktail: validated by the manufacturer in Western blot using lysates of human B cell lymphoma lines. Anti-HSP60: validated by the manufacturer in Western blot using lysates of pig kidney ESK-4 cell line. Anti-GAPDH: validated by the manufacturer in Western blot using cell lysates from mice and rat. Anti-Beta-actin: validated by the manufacturer in Western blot using lysates of human Hela cell line. Anti-CD3: validated by the manufacturer using Immunohistochemical staining of human T lymphocytes. Anti-CD4: validated by the manufacturer in Flow cytometry using Human peripheral blood lymphocytes. Anti-CD8: validated by the manufacturer in Flow cytometry using Human peripheral blood lymphocytes. Anti-CD27: validated by the manufacturer in Flow cytometry using Human peripheral blood lymphocytes. Anti-CD45RA: validated by the manufacturer in Flow cytometry using Human peripheral blood lymphocytes. Anti-CD25: validated by the manufacturer in in Flow cytometry using Human peripheral blood lymphocytes.

Eukaryotic cell lines

Policy information about cell lines and Sex and Gender in Research

Cell line source(s)

Jurkat T cells (Clone E6-1, (ATCC) TIB-152), HEK-293((ATCC), CRL-1573), Hela((ATCC), CRM-CCL-2) were purchased from ATCC originally and maintained in the lab as recommended by the vendor.

Authentication

No authentication has been used for this study.

Mycoplasma contamination

All cell lines used in the study tested negative for mycoplasma.

Commonly misidentified lines
(See ICLAC register)

No commonly misidentified cell lines were used in the study.

Plants

Seed stocks

Report on the source of all seed stocks or other plant material used. If applicable, state the seed stock centre and catalogue number. If plant specimens were collected from the field, describe the collection location, date and sampling procedures.

Novel plant genotypes

Describe the methods by which all novel plant genotypes were produced. This includes those generated by transgenic approaches, gene editing, chemical/radiation-based mutagenesis and hybridization. For transgenic lines, describe the transformation method, the number of independent lines analyzed and the generation upon which experiments were performed. For gene-edited lines, describe the editor used, the endogenous sequence targeted for editing, the targeting guide RNA sequence (if applicable) and how the editor was applied.

Authentication

Describe any authentication procedures for each seed stock used or novel genotype generated. Describe any experiments used to assess the effect of a mutation and, where applicable, how potential secondary effects (e.g. second site T-DNA insertions, mosiacism, off-target gene editing) were examined.

Flow Cytometry

Plots

Confirm that:

☒ The axis labels state the marker and fluorochrome used (e.g. CD4-FITC).

☒ The axis scales are clearly visible. Include numbers along axes only for bottom left plot of group (a 'group' is an analysis of identical markers).

☒ All plots are contour plots with outliers or pseudocolor plots.

☒ A numerical value for number of cells or percentage (with statistics) is provided.

Methodology

Sample preparation	PBMCs were thawed and washed in complete RPMI media before resting at 37 degree for 1 hour. PBMCs were stained with live/dead/proliferation dyes before washing and staining with fluorochrome-conjugated antibodies in FACS buffer. PBMCs were washed and filtered before data acquisition. Further details are provided in the method section.
Instrument	BD Celesta; BD FACS Aria Fusion.
Software	Data was acquired using FACS Diva and analyzed in FlowJo v.10 (BD)
Cell population abundance	For all populations of interest, a minimum of 3,000 cells were collected.<br>CD4+ T cell frequencies in lymphocyte gate (average)= 48.5% (naive 40.5%, memory 27.3%)<br>CD8+ T cell frequencies in lymphocyte gate (average)= 23.1% (naive 23%, memory 15.9%)
Gating strategy	For all experiments, cell events selected from the FSC/SSC plot were gated as singlets before live/dead discrimination. Human T cells were sorted according to the strategies presented in Supplementary Figures SIE. Gates for all populations referred to in the text are shown in the figures. Positive events for a given antigen were defined according to FMO/negative controls and cell types known to not express the antigen in pilot experiments and prior publications.

☒ Tick this box to confirm that a figure exemplifying the gating strategy is provided in the Supplementary Information.
